# Recent efforts to elucidate the scientific validity of animal-based drug tests by the pharmaceutical industry, pro-testing lobby groups, and animal welfare organisations

**DOI:** 10.1186/s12910-019-0352-3

**Published:** 2019-03-01

**Authors:** Jarrod Bailey, Michael Balls

**Affiliations:** 1 Cruelty Free International, 16a Crane Grove, London, N7 8NN UK; 2 Faculty of Medicine and Life Sciences, University of Nottingham, Nottingham, NG7 2UH UK

**Keywords:** Animal testing, Translational, Nonclinical, Clinical, Safety, Concordance

## Abstract

**Background:**

Even after several decades of human drug development, there remains an absence of published, substantial, comprehensive data to validate the use of animals in preclinical drug testing, and to point to their predictive nature with regard to human safety/toxicity and efficacy. Two recent papers, authored by pharmaceutical industry scientists, added to the few substantive publications that exist. In this brief article, we discuss both these papers, as well as our own series of three papers on the subject, and also various views and criticisms of lobby groups that advocate the animal testing of new drugs.

**Main text:**

We argue that there still remains no published evidence to support the current regulatory paradigm of animal testing in supporting safe entry to clinical trials. In fact, the data in these recent studies, as well as in our own studies, support the contention that tests on rodents, dogs and monkeys provide next to no evidential weight to the probability of there being a lack of human toxicity, when there is no apparent toxicity in the animals.

**Conclusion:**

Based on these data, and in particular on this finding, it must be concluded that animal drug tests are therefore not fit for their stated purpose. At the very least, it is now incumbent on—and we very much encourage—the pharmaceutical industry and its regulators to commission, conduct and/or facilitate further independent studies involving the use of substantial proprietary data.

## Background

Animal testing has been central to pre-clinical drug development for several decades, yet there remains no substantial, robust, published evidence that this has a scientific basis—i.e. that these tests are reliably predictive of human responses, both with respect to efficacy and toxicity/safety. With specific regard to toxicity, there are some analyses in the scientific literature (see, for example, [1–8]), but these are relatively few and limited, and with caveats: this must be considered perplexing, given the controversial nature of animal tests from an ethical perspective, and it also impacts significantly on human health and wellbeing.

Because of this, in 2013, we authored the first of a series of three papers (published in 2013–2015), which analysed publicly-available toxicity data on the use of animals in testing new drugs intended for human use [9–11]. These studies were ground-breaking, in that — in the face of a paucity of similar analyses (certainly comprehensive, robust and statistically appropriate ones) by the pharmaceutical industry — they constituted, to our knowledge, the most comprehensive published analyses of this kind to date, based on the largest database of animal and human toxicity studies yet compiled.

Briefly, we concluded, based on our thorough analyses that used the most-appropriate statistical methods, that the preclinical testing of pharmaceuticals in animals could not be justified on scientific grounds, as well as on ethical grounds. This position was based on the salient finding that the *absence* of toxicity in animals (dogs, rats, mice and rabbits and monkeys) provides essentially no insight into the likelihood of a similar lack of toxicity in humans: the former contributes no, or almost no, evidential weight in relation to the latter. Quantitatively, if, for example, a new drug has (based on prior information, such as similarity to other drugs, data from in vitro or in silico tests, and so on) a 70% chance of not being toxic in humans, then a negative test in any of these five species will increase this probability to an average of just 74%. The most controversial species, dogs and monkeys — the use of which, as opinion polls show, the general public object to particularly strongly — were the least predictive for humans in this respect, raising the probability from 70% to just 72 and 70.4% respectively. Therefore, animal tests provide essentially no additional confidence in the outcome for humans, but at a great ethical, and financial, cost.

## Main text

### Responses to our analyses of animal drug/toxicology tests, and continued defence of animal drug testing

Following the publication of each of our three, complementary papers in 2013, 2014 and 2015, we wrote to dozens of representatives of pharmaceutical companies, regulators and other stakeholders, requesting feedback, thereby hoping to build on our work and open some dialogue on this important issue, with ethical implications for the animals used, as well as for human users of pharmaceuticals. Disappointingly, only scant responses were received, and almost all of them were formulaic, and polite, but not engaging. The Association of the British Pharmaceutical Industry (ABPI) voiced some concerns over various attributes of the data set we used [12], but our substantial, published response constituted a full rebuttal [13]. Perhaps belatedly, the UK’s National Centre for the 3Rs (NC3Rs)—despite its initially dismissive stance—announced in the summer of 2016 its own collaborative project with the ABPI, to analyse industry data [14] We naturally welcome this, providing, of course, that it is done transparently and objectively, and preferably with independent oversight. Its eagerly-awaited report was expected in late 2018, but still has not been announced at the time of writing.

In the meantime, some advocates of animal drug-tests have continued to argue that these tests have utility, by citing some of the few, previous reports suggesting that this might be the case. This must be addressed, because this conclusion is not supported by those papers. One of these reports [2], as we have already discussed in our work, did not estimate specificity, without which the evidential weight toward likelihood of human toxicity/non-toxicity provided by the animal models—which is precisely what we need to know—cannot be calculated. As the authors of the cited study themselves acknowledged, “A more complete evaluation of this predictivity aspect will be an important part of a future prospective survey.” Another such cited report [15] showed human predictability for some therapeutic areas to be over 90%—yet it also showed many other areas where results from animal studies failed to significantly correlate with human observations, which were overlooked. Importantly, this analysis also utilised Likelihood Ratios (LRs), and the author argued why this is superior and necessary— much as we did in our own papers. Our rationale for using LRs—in place at the inception of our analyses, before any data were analysed, and in common with the aforementioned study—was, simply, because LRs are much more appropriate and inclusive, incorporating sensitivity and specificity, both of which are necessary to derive the true value of the results of any test, and which are superior to Predictive Values (PVs), because they do not depend on the prevalence of adverse effects. We discussed this in detail in our papers, and others have specifically supported this approach [16].

### Other, recent published analyses of drug toxicology data

Two studies similar to our own have been published in the past year. Given our interest in this, and given the ethical and scientific importance of the issue, we wish to add to the discussion and debate, by highlighting areas with which we agree and that we welcome, but also some issues we have with those papers and their conclusions.

### Monticello et al.

A study not limited to, but relying on, PVs was very recently published by Monticello et al. in November 2017 [17]. While we welcome and appreciate the authors’ attempts to elucidate this controversial and opaque issue, we believe their conclusion that, “These results support the current regulatory paradigm of animal testing in supporting safe entry to clinical trials and provide context for emerging alternate models”, must be addressed.

In our opinion, there are several important caveats. Perhaps the most salient is that—while the authors report both PVs and LRs—they focus almost exclusively on Negative Predictive Value (NPV) to support their conclusion. This is puzzling, given the nature of these statistical metrics and their associated qualities and shortcomings, and especially so, given that the authors specifically discuss some of them before ultimately overlooking them. For instance, even though they admit that LRs “are not influenced by clinical positive prevalence” (which is why, some assert, they may be superior), this doesn’t prevent the authors going on to concentrate on the PVs, which *are* influenced by toxicity prevalence.

We, in our analyses, argued, in some detail, why LRs should be used in preference to PVs [9–11, 13], as mentioned above. There is plentiful support for this in the literature. In brief, experts assert that LRs are the “optimal choice”, are “more informative than PVs”, and are “the single most powerful indicator of diagnostic usefulness”, as they incorporate sensitivity and specificity, and are independent of prevalence, which *must* be taken into account to estimate the value of a test (see [18–24]).

Monticello et al.’s emphasis on a high NPV is accepted to be “…largely based on the low clinical positive prevalence observed in our database and in the literature, which can be attributed to the fact that compounds entering clinical development have typically cleared many safety hurdles via extensive in silico, in vitro, and in vivo lead optimization screening activities.” Yet, it seems that the authors overlook the contribution of these screening activities, when they conclude that it is not they, but the lack of toxicity in animal tests, which predicts a lack of toxicity clinically, to the degree that they support the current paradigm centred on animal testing. What also challenges their conclusion—even taking the authors’ stance and sidestepping the LRs to concentrate on the PVs—is that their calculated Positive PVs (PPVs) were relatively low (a reported mean of just 36%, even when the low-scoring ‘other’ organ category was excluded); the authors chose to report that there were two impressive values out of the 36 reported, for non-human primates (NHPs), in the nervous system and gastrointestinal categories. We must question how this can “support the current regulatory paradigm of animal testing”. Animal tests aren’t just purported to exist to “support safe entry to clinical trials” by predicting which drugs might *not* be toxic to humans—they are also purported to serve as an efficient means of detecting which drugs might be harmful.

When one examines the LRs in Monticello et al.’s analysis instead of the PVs (see our argument above), a clearer picture emerges. The reported inverse Negative LRs (iNLRs) are very low indeed—sometimes less than 1.0, and often barely greater than unity—which suggests that the animal tests are providing no evidential weight to the probability that a drug will show no toxicity in humans. This is precisely the salient finding we reported in our papers [9–11], and which underpins our argument that the animal tests are not fit for purpose. They report a mean iNLR of just 1.5–1.6, and a mean Positive LR (PLR) of 2.9. These are low LR values, which indicate that very little evidential weight is being provided by the animal tests to the probability of human toxicity/absence of toxicity. They also report similarly poor iNLRs for rodents, dogs and monkeys, as we found. In short, in many ways, they actually repeat and reinforce our findings, in accordance with their statement in section 2.7 of their Methods, that, “As a general rule, a test is considered ‘diagnostic’ in predicting a positive outcome when the LR+ is >10 or for predicting a negative outcome when the iLR- is > 10.” Of their 36 possible results, only two PLRs/LR+ met the authors’ acknowledged ‘diagnostic’ definition of a value of > = 10, and none of the iNLRs/iLR- did so. In fact, 30 of the iLR- values were < =2, with most of these in or around unity; i.e. they provided no evidential weight at all. In other words, by the definition and criteria that they cite, the animal tests, based on their data and their analysis, cannot be considered to be diagnostic/predictive.

We appreciate that the authors acknowledge some important points about this area of science generally, as well as some limitations of their study. As we did in our own work, they report “limited” efforts to analyse the value of animal tests in the past, and accept they are based on “historical precedence” and an assumption of value. With regard to their analysis, they accept that their data involved just 182 drugs (compared to our > 3200, for example); they looked only at animal test/Phase I concordance, and didn’t include later phase clinical trials, in which more drugs will fail. Their study also used few, broad categories for adverse drug reactions (ADRs), which favours their hypothesis compared to more, and more stringent, classifications; and they combined mice and rats as ‘one effective species’, even though mice and rats often show significant differences in toxicity [11]. Finally, they reported no conflicts of interest, but thanked almost 20 biopharmaceutical companies in their acknowledgements, and have affiliations to nine companies. While we do not suggest any impropriety, some might argue that they could have an interest in justifying their industry’s and companies’ historic and current use of animals in drug testing.

### Clark and Steger-Hartmann

This was an analysis of more than 3000 drugs, based on data in Elsevier’s comprehensive PharmaPendium database [25, 26]. The authors took a similar approach to our own, by using LRs to determine the diagnostic power of tests in animals to inform human toxicity, as well as concluding that their study confirmed our own salient finding: *“…the lack of these [adverse] events in nonclinical* [animal] *studies was found to not be a good predictor of safety in humans, thus partly confirming the findings of Bailey et al. (2014).* [citing one of our series of three papers]*”*.

Confirmation of our salient finding is of the utmost importance for two reasons. First, though we sought no validation of our own approach and publications, but have always had the utmost confidence in them, some stakeholders with opposing opinions on the value of animal-based drug testing were intent on denigrating our work. Secondly, no matter how well any animal test might predict human toxicity (hypothetically), it is the *absence* of toxicity in animals that is the critical factor for the progression of a new drug into clinical (human) trials. As we continue to argue, if animal tests fail in this crucial respect—as they appear to do—this not only means those tests are not fit for their overall purpose (identifying safe and effective human drugs), but this must have repercussions for the pharmaceutical industry and its regulators, and how they approach drug testing generally.

This paper also confirmed our other main finding, which suggested that adverse reactions in animal tests are, in fact, also likely to occur in humans (though, importantly, often not in a similar manner). Crucially, however, we have interpreted the consequences of this aspect differently. Both the authors of this paper, and ourselves, found this aspect to be very variable, with no clear pattern in terms of types of toxic effects or types of drugs. We therefore concluded that this cannot be considered particularly relevant or reliable. Clark and Steger-Hartmann, however, provided some examples of where animals did predict human toxicity, but did not show, or weigh these against, areas where this predictive aspect was lower, non-existent, or negative. Indeed, some of the examples they provided were only just over the statistical threshold they had themselves had set. Consequently, we believe that while both their data and our own data support their conclusion that, “The animal-human translation of many key observations is confirmed as being predictive”, they do *not* support their conclusion that their study “…confirmed the general predictivity of animal safety observations for humans”. This is compounded by very poorly predictive observations that can only be considered as serious, such as death, convulsions, movement disorders and liver disorders.

## Conclusions

The first salient point must be this: to determine the evidential weight provided by animal tests to the probability of human toxicity/non-toxicity of new drugs—which is the specific question that must be asked to determine the scientific value of these tests—it is LRs that must be used as the statistical metric, not PVs. We made the case for this in the series of three papers describing our own studies, and, prior to, and during, our analyses, we sought the advice of two professional and eminent European statisticians, and an experienced pharmaceutical consultant au fait with the matter [16], who concurred. We acknowledge that all statistical approaches have their advantages and disadvantages, and multiple approaches can be informative. Further, we appreciate that more-complex Bayesian modelling may be required to gain further insight into the matter in the future, for instance, in addition to fuller harm–benefit analyses and looking into specific pharmaceutical and toxicological areas. However, we believe that the evidence shows—as we mentioned above—that LRs are more informative, inclusive and valuable than PVs, at least when used on their own and as a first step in gauging how predictive animal testing might be for human toxicity [18–24].

This has not prevented some individuals/groups who defend animal-based drug testing from focusing on PVs and overlooking LRs, and, perhaps more seriously, omitting mention of the most conspicuous and pivotal finding of our studies. That is the second salient point, which is that the *absence* of toxicity in animals provides essentially no insight into the likelihood of a similar lack of toxicity in humans. As the absence of toxicity in animals is the critical factor for the progression of a new drug into clinical trials, this has extremely important implications for drug development and safety. Our analyses indicate that, if a drug appears safe in animals, it could very well still be toxic in humans. Thus, any claim that animal safety tests do a “good job” of predicting drug safety profiles, is without foundation. This has serious ethical implications that are of interest to the readers of this journal. Millions of animals are used in drug testing every year around the world, which can entail severe suffering, pain and death, which most people (at least in the UK, EU and USA) oppose, regardless of human benefit [27–31]. Suffering in animal drug testing is often severe and prolonged: animals used in chronic toxicity and carcinogenicity studies, for instance, receive the test substance at high doses, daily, seven days a week, for two years with no recovery periods [32], and The Organisation for Economic Co-operation and Development (OECD) [33] and the Nuffield Council on Bioethics [34] list the following as common conditions and clinical signs that may occur during such tests, which indicate that an animal is experiencing pain and/or distress, and suffering: Gasping, difficulty breathing, excess salivation and nasal discharge, tremor, changes in blood pressure, seizures, convulsions, coma, abnormal vocalization, aggression, diarrhoea, vomiting, bleeding from any orifice, oedema, abdominal rigidity, rectal or vaginal prolapse, swollen joints, and paralysis.

In addition, there are human ethical consequences. If animal testing of proposed new human drugs is not sufficiently predictive of human safety—or, as we argue at least in some respects, not predictive at all—then there is significant human suffering, pain and death, too, as science and drug development are not serving human drug users and sick people who are depending on the best science being conducted to develop much needed new drugs that are safe and effective. Drugs appearing to have no serious toxicity may go on to cause human harm either in clinical trials or, even worse, if they pass through clinical trials involving relatively limited numbers of people, are of limited duration and involve limited lifestyle circumstances and factors, and make it to market where they reach millions of users and may have to be withdrawn when their toxicity to humans is recognised. It is also acknowledged that drugs appearing to have serious toxicity in animal tests will not proceed to human trials, so drugs that may have been safe and effective in humans will have been lost.

We reiterate that we welcome any objective efforts to shed light on the value—or lack of value—of animal tests for drugs intended for human use. However, for all the reasons outlined above, we must contend that the two most recent publications discussed, while they have much merit, do not, as their authors conclude, “…support the current regulatory paradigm of animal testing in supporting safe entry to clinical trials and provide context for emerging alternate models” [17], or “confirm[ed] the general predictivity of animal safety observations for humans” [26].

In any case, prima facie, it seems clear that there is something gravely wrong with the way in which drugs are developed and tested: more than 90% of the drugs that appeared to be safe and effective in animals went on to fail in human trials between 2006 and 2015 [35, 36]. It has been claimed that this simply is ‘a reflection of normal design process’, but would this failure rate be thus described and acceptable for aeroplanes, car brakes, or nuclear power stations? When this process is putting people at risk—as is the case in drug development—this excuse cannot be valid. It is claimed that the absence of thousands of human deaths in Phase I clinical trials illustrates that animal testing is fit for purpose, yet this overlooks the precautionary and carefully monitored nature of these trials, which involve few individuals (typically 6–12), and the administration of small doses. Therefore, the fact that *any* unexpected deaths have occurred in Phase I trials may be considered alarming, but examples include: the TGN1412 (Northwick Park) trial in 2006 [37–42]; several deaths in a hepatitis drug trial (fialuridine) in 1993 [43, 44]; and Bial’s BIA 10–2474, which killed and hospitalised (via cerebral micro-bleeds) clinical trial volunteers [45–47]. It was subsequently shown that non-animal tests could, and would, have detected these events, at least for TGN1412 [48, 49], fialuridine [50] and BIA 10–2474 [51], if they had been more widely used. In 2016, five unexpected deaths were reported of cancer patients in a CAR-T immunotherapy trial for Juno Therapeutics, attributed at the time to an interaction between genetically-engineered cells being infused into the patients and a co-administered chemotherapy drug. [52] A study of US Food and Drug Administration (FDA) clinical hold orders, which may be enforced during drug development, if an unreasonable risk is perceived for participating subjects, revealed that, between 2008 and 2014, 29 such holds had been actioned; seven for unexpected deaths, and nine involving unexpected target organ damage [53]. Claims that most drug failures are *not* for reasons of toxicity or efficacy undetected in preclinical studies, are false [54–56].

The ethical implications of persisting with the status quo of animal-oriented drug testing and development are therefore clear. It is undoubtedly time for a serious, large-scale, industry-wide consideration of the necessity of animal drug testing, involving all the stakeholders—regulators, scientists, politicians, developers of alternative testing methods, and so on. This must entail a *critical* attitude, asking what is wrong with the animal tests, and identifying areas in which they are not performing, instead of seeking to justify those tests by identifying particular pharmaceutical or toxicological areas in which they do have some human-predictive power and passively accepting that that is a sufficient justification for their general application. This must also involve a deep deliberation on what all the myriad alternative methods, human, in vitro and in silico can provide when used together in intelligent strategies, which are increasingly capable, astounding, and of course, human relevant (see, for example, the proceedings of the 10th World Congress on Alternatives and Animal Use in the Life Sciences [57], and [58–61]). Instead of expecting perfection from them, highlighting where they fall short, and applying standards of validation to them that the animal tests have never met, and could never meet, their application must be weighed against the ethical cost of animal testing—the suffering, pain and death involved for millions of animals every year—and the human ethical cost of developing toxic and harmful medicines, and of missing medicines that would have been safe and effective, but that were terminated due to serious animal toxicities which may not have been relevant to humans.

## Abbreviations

ABPI: Association of the British Pharmaceutical Industry
ADR: Adverse drug reaction
FDA: US Food and Drug Administration
LR: Likelihood ratio (PLR - Positive LR; iNLR - inverse Negative LR)
NC3Rs: National Centre for the 3Rs
NHP: Nonhuman primate
PV: Predictive value (PPV - Positive PV; NPV - Negative PV)
